# T1ρ relaxation mapping in osteochondral lesions of the talus: a non-invasive biomarker for altered biomechanical properties of hyaline cartilage?

**DOI:** 10.1186/s41747-024-00488-4

**Published:** 2024-07-24

**Authors:** Balázs Bogner, Markus Wenning, Pia M. Jungmann, Marco Reisert, Thomas Lange, Marcel Tennstedt, Lukas Klein, Thierno D. Diallo, Fabian Bamberg, Hagen Schmal, Matthias Jung

**Affiliations:** 1 Department of Diagnostic and Interventional Radiology, University Medical Center Freiburg, Faculty of Medicine, University of Freiburg, 79106 Freiburg, Germany; 2 Berta-Ottenstein-Programme, Faculty of Medicine, University of Freiburg, Freiburg, Germany; 3 Department of Orthopedics, BDH Klinik Waldkirch, 79283 Waldkirch, Germany; 4 Praxis Drescher-Eberbach-Wenning, Orthopedic Surgeons, 79100 Freiburg, Germany; 5 Division of Medical Physics, Department of Diagnostic and Interventional Radiology, University Medical Center Freiburg, Faculty of Medicine, University of Freiburg, 79106 Freiburg, Germany; 6 Department of Stereotactic and Functional Neurosurgery, University Medical Center Freiburg, University of Freiburg, Faculty of Medicine, University of Freiburg, 79106 Freiburg, Germany; 7 Department of Orthopedic and Trauma Surgery, University Medical Center Freiburg, University of Freiburg, Faculty of Medicine, University of Freiburg, 79106 Freiburg, Germany

**Keywords:** Ankle joint, Biomarkers, Hyaline cartilage, Magnetic resonance imaging, Talus

## Abstract

**Background:**

To evaluate T1ρ relaxation mapping in patients with symptomatic talar osteochondral lesions (OLT) and healthy controls (HC) at rest, with axial loading and traction.

**Methods:**

Participants underwent 3-T ankle magnetic resonance imaging at rest and with 500 N loading and 120 N traction, without axial traction for a subcohort of 17/29 HC. We used a fast low-angle shot sequence with variable spin-lock intervals for monoexponential T1ρ fitting. Cartilage was manually segmented to extract T1ρ values.

**Results:**

We studied 29 OLT patients (age 31.7 ± 7.5 years, 15 females, body mass index [BMI] 25.0 ± 3.4 kg/m^2^) and 29 HC (age 25.2 ± 4.3 years, 17 females, BMI 22.5 ± 2.3 kg/m^2^. T1ρ values of OLT (50.4 ± 3.4 ms) were higher than those of intact cartilage regions of OLT patients (47.2 ± 3.4 ms; *p* = 0.003) and matched HC cartilage (48.1 ± 3.3 ms; *p* = 0.030). Axial loading and traction induced significant T1ρ changes in the intact cartilage regions of patients (loading, mean difference -1.1 ms; traction, mean difference 1.4 ms; *p* = 0.030 for both) and matched HC cartilage (-2.2 ms, *p* = 0.003; 2.3 ms, *p* = 0.030; respectively), but not in the OLT itself (-1.3 ms; *p* = 0.150; +1.9 ms; *p* = 0.150; respectively).

**Conclusion:**

Increased T1ρ values may serve as a biomarker of cartilage degeneration in OLT. The absence of load- and traction-induced T1ρ changes in OLT compared to intact cartilage suggests that T1ρ may reflect altered biomechanical properties of hyaline cartilage.

**Trial registration:**

DRKS, DRKS00024010. Registered 11 January 2021, https://drks.de/search/de/trial/DRKS00024010.

**Relevance statement:**

T1ρ mapping has the potential to evaluate compositional and biomechanical properties of the talar cartilage and may improve therapeutic decision-making in patients with osteochondral lesions.

**Key Points:**

- T1ρ values in osteochondral lesions increased compared to intact cartilage.
- Significant load- and traction-induced T1ρ changes were observed in visually intact regions and in healthy controls but not in osteochondral lesions.
- T1ρ may serve as an imaging biomarker for biomechanical properties of cartilage.

**Graphical Abstract:**

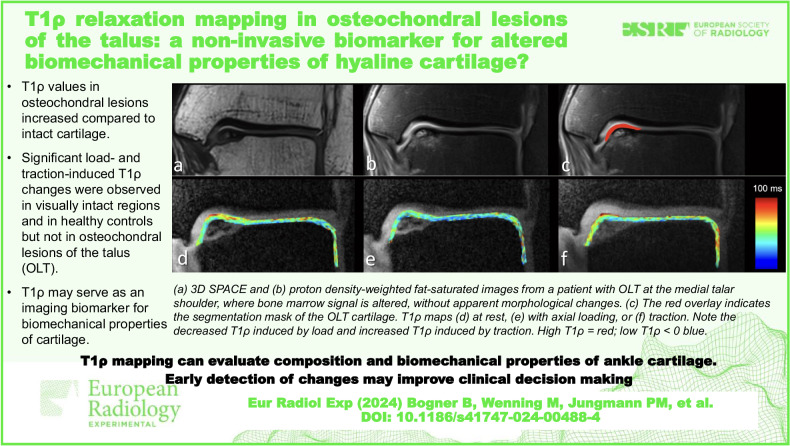

## Background

Osteochondral lesions of the talus (OLT) refer to injuries involving talar articular cartilage and the subchondral bone [1]. OLTs frequently occur in physically active individuals and are reported in up to 73% of traumatic ankle injuries [2]. OLTs appear to be a significant joint-related risk factor for early osteoarthritis (OA) of the upper ankle joint, severely limiting athletic performance and potentially causing significant loss of quality of life in a young and athletic population [3, 4]. Timely therapeutical intervention to restore cartilage tissue may prevent the development of OA or delay its progression [5].

The underlying pathophysiology of OLTs is not fully understood. Repeated microtrauma to the articular cartilage as a possible causative factor may lead to disruption of the cartilage collagen network and secondary subchondral bone necrosis. Further, significant traumatic injury to the subchondral bone in the ankle may result in subsequent damage to the overlying articular talar cartilage. Regardless of the exact mechanism, chondral injuries are associated with a gradual decrease in cartilage proteoglycan content and an increase in water content due to disruption of the extracellular matrix [6, 7].

The detection of early changes in the cartilage tissue of OLT is a prerequisite for appropriate treatment [8]. Magnetic resonance imaging (MRI) enables noninvasive evaluation of the cartilage and subchondral bone and is, therefore, a valuable tool to assess OLT and related OA as a whole organ pathology [9]. However, conventional MRI techniques lack sensitivity in detecting subtle changes in the cartilage matrix which may be associated with early OA [10]. In contrast, quantitative MRI techniques, such as T2 relaxation time measurements and T1ρ relaxation time measurements provide information about compositional changes of the hyaline cartilage matrix in patients with OLT. T1ρ mapping allows for noninvasive quantitative assessment of biochemical changes of the cartilage matrix that are sensitive to early stages of OA which are not visible on morphological MRI [11, 12]. Since ultrastructural changes may correlate with the biomechanical performance of the hyaline cartilage, T1ρ mapping can be useful to noninvasively examine cartilage weight-bearing properties. Recent studies indicate that changes in T1ρ relaxation times may serve as an imaging biomarker of altered biomechanical properties of hyaline cartilage in patients with chronic ankle instability [13–15]. By applying *in situ* mechanical stress during the MRI examination the biomechanical and biochemical properties can be examined simultaneously [16–20]. Since chronic ankle instability can cause OLTs and eventually OA [21, 22], it is of high clinical interest to assess potential biochemical and biomechanical changes derived from T1ρ mapping in these patients.

The purpose of this study was to assess T1ρ relaxation times in patients with OLT and healthy control subjects at rest, with axial loading and traction. We hypothesized that loading- and traction-induced changes in T1ρ relaxation time may identify altered biomechanical properties of articular cartilage in patients with OLT.

## Methods

The local Institutional Review Board approved the study which was prospectively registered at the German Clinical Trials Register (Trial registration: DRKS, DRKS00024010, January 11, 2021; https://drks.de/search/de/trial/DRKS00024010). Informed written consent was obtained from all individual participants included in the study. All procedures involving human participants were performed according to the ethical standards of the institutional and national research committee and to the Declaration of Helsinki in its current form.

### Subjects

Inclusion criteria for healthy controls (HC) were the absence of symptoms at the ankle joint, stable ankles during clinical examination, no ankle sprain or any other traumatic ankle injury within the last two years, and a normal score according to the Cumberland Ankle Instability Tool-CAIT [23]. HC aged 18–50 years were randomly recruited from a community sample of students and employees working at the university. Patients with OLT were recruited from an outpatient clinic specializing in the treatment of ankle injuries. There, the OLT diagnosis was based on clinical examination and confirmed by standard clinical MRI. After inclusion in this study, the OLT patients underwent a standardized morphological and quantitative MRI for this study (see below).

**Fig. 1:**
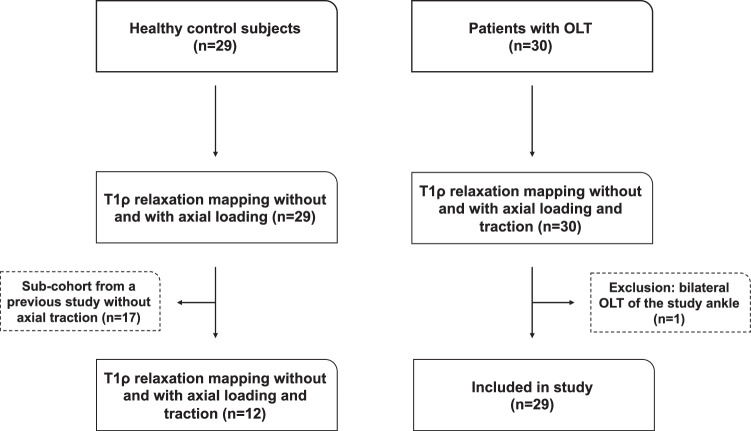
Flow diagram of the study population. OLT, Osteochondral lesion of the talus

Inclusion criteria for patients were OLT-related symptoms such as load-induced pain or swelling for at least 6 months before enrollment. In addition, all patients had a history of ankle sprain not closer than 6 months before MRI examination. General exclusion criteria were age < 18 years, previous ankle surgery, traumatic ankle injury within the last 6 months, neurologic disorders, diabetes mellitus, and MRI contraindications. A subcohort of the HC group (17/29; 58.4%) included in this study was recruited from our previous study on T1ρ mapping for chronic ankle instability (https://pubmed.ncbi.nlm.nih.gov/35611813/) [15]. This study focused on ankle instability and did not include any individuals with osteochondral lesions of the ankle joint and did not report on *in situ* axial traction. For this subcohort, no quantification of T1ρ values under *in situ* axial traction was available. Therefore, only 12 HC were included in the statistical analysis involving the group and pairwise comparisons of T1ρ changes after *in situ* traction. One patient was excluded from further analysis after initial MRI acquisition due to bilateral OLT (Fig. 1).

### MRI acquisition

MRI of the ankle was performed using a 3-T scanner (Magnetom Trio, Siemens Healthineers, Erlangen, Germany), with a dedicated 8-channel multi-purpose coil (NORAS, MRI products, Höchberg, Germany) for signal reception. Patients with OLT and HC prospectively underwent MRI of the ankle at rest and with *in situ* axial loading of 500 N and traction of 120 N.

For morphological evaluation, we acquired:

- an isotropic three-dimensional proton density turbo spin echo sequence (Sampling Perfection with Application optimized Contrast using different flip angle Evolution−SPACE), with the following parameters: repetition time 900 ms; echo time 41 ms; flip angle 130°; voxel size 0.5 × 0.5 × 0.6 mm^3^, phase encoding direction right-left; field of view 150 × 150 mm^2^; bandwidth 504 Hz/pixel; total acquisition time 4:55 min:s; and
- a coronal proton density turbo spin-echo with fat-suppression with the following parameters: repetition time 3.000 ms; echo time 32 ms; flip angle 160°; voxel size 0.4 × 0.3 × 3.0 mm; phase encoding direction anterior-posterior; field of view 150 × 150 mm^2^; bandwidth 248 Hz/pixel; total acquisition time 3:44 min:s.

For T1ρ relaxation mapping we acquired a three-dimensional fast low-angle shot sequence with the following parameters: echo train duration 2.380 ms; repetition time 9.3 ms; echo time 4.62 ms; flip angle 10°; voxel size 0.3 × 0.3 × 2.4 mm; matrix size 256 × 256; in-plane resolution 0.3 mm; 12 partitions; readout direction right-left; phase encoding direction anterior-posterior and feet-head; field of view 80 × 80 mm^2^; bandwidth 330 Hz/pixel; total acquisition time 5:00 min:s. For monoexponential pixel-by-pixel T1ρ fitting, a spin-lock frequency of 500 Hz was used with five different spin-lock durations (τ = 0/10/20/30/40 ms). To avoid artifacts due to magnetic field inhomogeneities (ΔB_0_), we implemented a ΔB_0_ and B_1_ insensitive spin-lock pulse cluster described by Witschey et al [24]. The preparation interval τ was divided into two periods with opposite spin-lock B_1_ phase, separated by a 180° pulse for ΔB_0_ refocusing (90°x-τ/2y-180°y-τ/2-y-90°-x). Fat saturation was applied between the spin-lock preparation interval and the fast low-angle shot module. The reproducibility of the T1ρ mapping sequence used in this study was previously evaluated in a previous study. T1ρ mapping was performed on two healthy volunteers, both at rest and under *in situ* axial loading of 200 N and 400 N, with a time interval of 6 months and 2 weeks between the measurements, respectively. T1ρ mapping results showed high test-retest reproducibility between different time points and under different mechanical conditions [25].

**Fig. 2:**
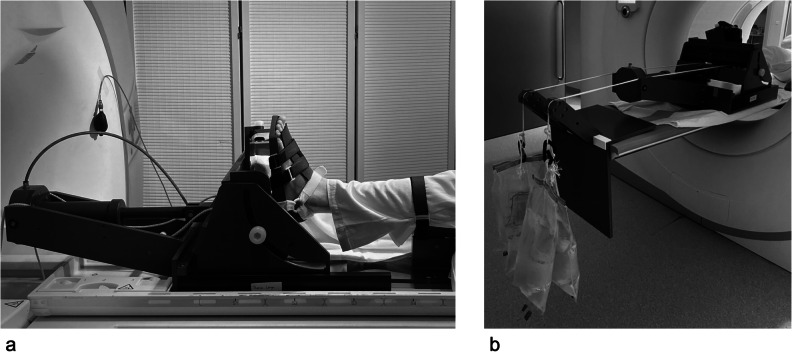
Experimental setup for axial loading and traction of the ankle joint during acquisition. Mock-up experimental setup for axial (**a**) loading and (**b**) traction of the ankle joint. The custom-built arthrometer enables stable ankle positioning in a neutral position of 90° dorsiflexion. Note that the lower leg was fastened to a leg holder and a strap above the knee to prevent motion-induced artifacts. **a** Axial load was applied using a pneumatic loading system. **b** For axial traction, two 6-liter water-filled bags were connected to an ankle distractor foot strap through a cord pulley system

All ankle MRI scans were performed (i) at rest, (ii) with *in situ* axial loading of 500 N, and (iii) with traction of 120 N with the examined ankle in a neutral position at 90° dorsiflexion. For both, *in situ* axial loading and traction, a custom-made arthrometer was used (Fig. 2). A minimum of 25 min of ankle rest was allowed before quantitative imaging due to the acquisition of morphological sequences and patient information. Before *in situ* axial loading, a resting period of 15 min was obtained. Before axial traction, there was an additional resting period of 10 min during the setup of the traction arthrometer. For *in situ* axial loading, 500 N was applied using a pneumatic loading system to generate a significant loading effect without exciding the weight bearing on the ankle during single-leg stance. For axial traction, an ankle distractor foot strap was fixed around the talus using a cord and pulley system, connected to two 6-liter water-filled bags. During the MRI acquisition, subjects were fixed to the scanner bed with a pelvic belt. In addition, the lower leg was fastened to a leg holder and a strap above the knee to maintain the knee in full extension and to prevent motion-induced artifacts from mechanical loading and traction.

### Image analysis

For morphological evaluation, all images were evaluated on a dedicated workstation (Dedalus Healthcare Group, Bonn, Germany). The location of the OLT was noted and classified as medial and lateral. To determine the size of the OLTs, maximum coronal and sagittal diameters were measured. The size of the OLT was calculated using the ellipse formula described by Choi et al [26]. Further, OLTs were graded according to Anderson et al by a board-certified radiologist with 10 years of experience in musculoskeletal radiology (T.D.D.) [27].

Quantitative MRI data was evaluated using a web-based medical image analysis platform (Nora Medical Imaging Platform, Freiburg; (available free of charge online: https://www.nora-imaging.org/demo/index.php?viewer). T1ρ maps were calculated by a mono-exponential pixel-by-pixel fit according to the following signal equation:

$${{{{\rm{S}}}}}\left({{{{\rm{\tau }}}}}\right)={{{{\rm{S}}}}}0\, \cdot \, \exp \left(-\frac{{{{{\rm{\tau }}}}}}{{{{{\rm{T}}}}}1{{{{\rm{\rho }}}}}}\right)$$

where S(τ) is the measured signal intensity of the image for a given spin-lock duration τ and S_0_ is the signal intensity for τ = 0.

For T1ρ relaxometry, twelve coronal slices were reconstructed. To avoid partial volume artifacts due to the presence of potentially large amounts of synovial fluid at the peripheral regions of the cartilage layer, one anterior and one posterior slice were excluded from further analysis. Visually intact talar cartilage was manually segmented on the remaining ten coronal slices by two readers (B.B., 4 years of experience; M.T., 2 years of experience), carefully avoiding the corticalis, synovial fluid, and the OLT area. Subsequently, each volume of interest was divided into a medial and lateral subset at the midpoint of the superior articular surface of the talus according to the maximum curvature. The OLT cartilage area was manually annotated in a separate volume of interest. To avoid bias due to regional differences in T1ρ values in the talar cartilage, we performed a regional matching to compare intact cartilage regions from OLT patients with cartilage from HC subjects [28]. T1ρ values obtained from talar cartilage regions contralateral to the OLT location were compared (Supplementary Fig. S1). Both readers were trained in cartilage segmentation by one of the senior authors (P.M.J., 15 years of experience). T1ρ data sets from ten independent subjects were analyzed by both readers (B.B., 4 years of experience; M.T., 2 years of experience) with at least 6 months between the two readings to assess interobserver agreement. In addition, to assess intraobserver agreement, measurements were repeated by one reader (B.B., 4 years of experience) after a minimum of four weeks to maintain retest independence.

For quality control, all segmentations were independently validated and adjusted where necessary by a board-certified radiologist (P.M.J., 15 years of experience).

### Statistical analysis

Median T1ρ values were extracted from each volume of interest. Median T1ρ values of all volumes of interest showed normal distribution in quantile-quantile plots. Median T1ρ values of the OLT cartilage, visually intact cartilage regions of OLT patients, and matched cartilage of HC were compared using one-way analysis of variance (ANOVA) and post-hoc *t*-tests (Bonferroni-Holm corrected to avoid alpha error accumulation due to multiple testing). Changes in T1ρ relaxation times after axial loading and traction were compared using one-way ANOVA and post-hoc paired *t*-tests (Bonferroni-Holm). Due to unequal sample sizes, median T1ρ values of OLT cartilage obtained from different OLT grades were compared using the Kruskal-Wallis test and post-hoc Wilcoxon and paired Wilcoxon tests (Bonferroni-Holm corrected). To assess the intraobserver and interobserver agreement of image segmentation, we calculated the intraclass correlation coefficient (ICC, two-way mixed effect ANOVA). We used the following scale to interpret the ICC results: poor (ICC < 0.5), moderate (ICC 0.5–0.7), good (ICC 0.7–0.9), or excellent (ICC > 0.9) reproducibility [29]. The *p*-values lower than 0.05 were considered to be statistically significant. All statistical analyses were performed using R statistics (R-4.2.2 – R Core Team, https://www.r-project.org/).

## Results

### Patients characteristics

A total of 29 OLT patients (age 31.7 ± 7.5 years; body mass index (BMI) 25.0 ± 3.4 kg/m^2^; 15 females, 51.7%) and 29 HC (age 25.2 ± 4.3 years; BMI 22.5 ± 2.3 kg/m^2^; 17 females, 58.6%) were included in this study. Of the 29 OLT lesions, 24 (82.8%) were located on the medial talar shoulder. The OLT area was 108.8 ± 57.5 mm^2^ (mean ± standard deviation). The majority of the OLTs (13/29, 44.8%) were graded as grade 3; the remaining were graded as grade 1 (*n* = 3; 10.3%), grade 2a (*n* = 7; 24.1%), grade 2b (*n* = 4; 13.8%), and grade 4 (*n* = 2; 6.9%). Patients’ characteristics are described in Table 1.

**Table 1:** Patient characteristics

	Healthy controls	OLT
Group size	29	29
Sex (males/females)	12/17	15/14
Age (years)	25.2 ± 4.3	31.7 ± 7.5
Body mass index (kg/m^2^)	22.5 ± 2.3	25.0 ± 3.4
OLT location (medial/lateral)	−	24/5
OLT area (mm^2^)	−	108.8 ± 57.5
OLT grade (1, 2a/b, 3, 4)	−	3/7/4/13/2
Cumberland Ankle Instability Tool	30	15.47 ± 6.73

Values are given as mean values ± standard deviation

*CAIT* Cumberland ankle instability tool score, *OLT* Osteochondral lesion of the talus

### Intraobserver and interobserver agreement

We found good to excellent intraobserver agreement for HC at rest (ICC 0.845), with *in situ* axial loading (ICC 0.990) and traction (ICC 0.947). For cartilage segmentation in the visually intact cartilage regions of OLT patients, the ICC was 0.973 at rest, 0.898 with axial loading, and 0.933 with traction. Furthermore, in the OLT cartilage, ICC was 0.956 at rest, 0.986 with axial loading, and 0.901 with traction.

Also, the interobserver agreement was found to be good to excellent for cartilage segmentation in HC, at rest (ICC 0.807), under *in situ* axial loading (ICC 0.973), and under traction (ICC 0.788). Similarly, in the visually intact cartilage regions of OLT patients, interobserver agreement was good to excellent, at rest (ICC 0.929), with *in situ* axial loading (ICC 789), and with traction (ICC: 832). In the cartilage layer covering the OLT, the ICC was 0.909 at rest, 0.928 with *in situ* axial loading, and 0.864 with traction.

### T1ρ values of OLT cartilage *versus* visually intact cartilage regions

**Fig. 3:**
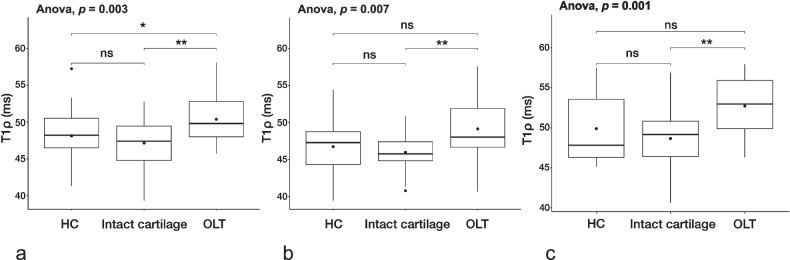
T1ρ relaxation mapping in OLT cartilage *versus* visually intact cartilage regions. **a** T1ρ values in the OLT cartilage were significantly higher compared to visually intact cartilage regions. **b** After axial loading and (**c**) traction, T1ρ values in the OLT cartilage remained significantly higher than the intact cartilage regions in OLT patients, but not those of matched HC cartilage. There was no significant difference between visually intact cartilage regions in OLT patients and matched HC cartilage (**a**–**c**). Boxplots: the middle line represents the median; the upper and lower ends of the box represent the 75th and 25th percentiles, respectively. The black dot in the box represents the mean. *p < 0.050; **p < 0.010; ns, Not significant. HC, Healthy controls; OLT, Osteochondral lesion of the talus

At rest, we found significantly higher T1ρ values in the OLT cartilage (50.4 ± 3.4 ms) compared to visually intact cartilage regions of OLT patients (47.2 ± 3.4 ms; *p* = 0.003) and matched cartilage of HC (48.1 ± 3.3 ms; *p* = 0.030; Fig. 3). There was no significant difference in the OLT cartilage between different OLT grades (*p* = 0.870).

There was no significant difference between the visually intact cartilage regions of OLT patients and the matched cartilage of HC (*p* = 0.280). Furthermore, no significant regional differences in the talar cartilage were found in HC (*p* = 0.077). After axial loading, T1ρ values in the OLT cartilage (49.2 ± 4.33 ms) remained significantly higher than in the visually intact cartilage regions of OLT patients (46.0 ± 2.6 ms; *p* = 0.008), but not compared to the matched cartilage of HC (46.8 ± 3.8; *p* = 0.090). Similarly, after traction, OLT cartilage T1ρ values (52.7 ± 3.8 ms) were significantly higher than those of visually intact cartilage regions of OLT patients (48.7 ± 3.9 ms; *p* = 0.001), but not compared to the matched cartilage of HC (49.9 ± 4.4; *p* = 0.140). During axial loading, T1ρ values of grade 1 OLTs (39.14 ± 4.94 ms) were significantly lower than those of grade 2 (51.15 ± 5.13 ms; *p* = 0.042) and grade 3 OLTs (48.24 ± 2.15 ms; *p* = 0.042), but not grade 4 OLTs (49.15 ± 4.09 ms; *p* = 0.800). There were no significant differences between grade 2, 3, and 4 OLTs (grade 2 *versus*. 3; *p* = 0.370, grade 2 *versus* 4; *p* = 0.727, and grade 3 *versus* 4; *p* = 0.606). During axial traction, there were no significant differences in T1ρ values between different OLT grades (grade 1 *versus* 2; *p* = 0.769, grade 1 *versus* 3; *p* = 0.937, and grade 1 *versus* 4; *p* = 0.999, grade 2 *versus* 3; *p* = 0.387, grade 2 *versus* 4; *p* = 0.769, and grade 3 *versus* 4; *p* = 0.909).

Both, after axial loading and traction, there was no significant difference between visually intact cartilage regions of OLT patients and matched cartilage of HC (*p* = 0.420 and *p* = 0.410, respectively).

### Change in T1ρ values after loading and traction

**Fig. 4:**
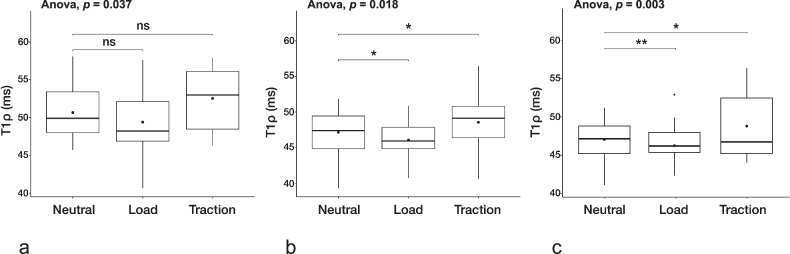
Change in T1ρ values after loading and traction. T1ρ relaxation values at rest and with axial loading and traction (**a**) in the OLT cartilage, (**b**) in visually intact cartilage regions of OLT patients, and (**c**) in matched HC cartilage. **a** Both loading and traction did not induce significant T1ρ changes in the OLT. Axial loading significantly decreased T1ρ relaxation times in visually intact cartilage regions of (**b**) OLT patients and (**c**) matched HC cartilage, while traction led to a significant increase. Boxplots: middle line represents the median; the upper and lower ends of the box represent the 75th and 25th percentiles, respectively. The black dot in the box represents the mean. *p < 0.050; **p < 0.010; ns, Not significant. HC, Healthy controls; OLT, Osteochondral lesion of the talus

**Fig. 5:**
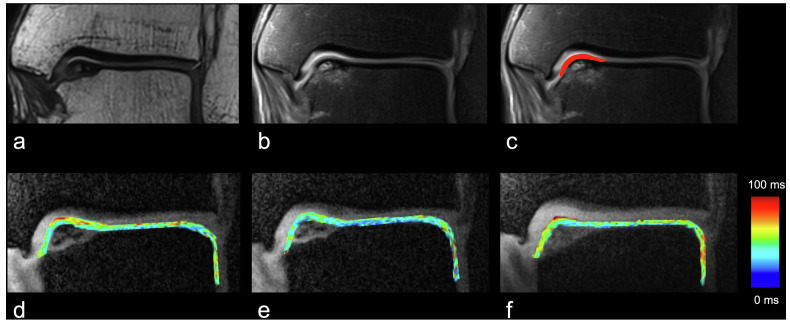
Representative morphological images and T1ρ maps under different mechanical conditions. Coronal (**a**) 3D SPACE (see text for details on the sequence) and (**b**) proton density-weighted fat-saturated images from a patient with OLT located at the medial talar shoulder. Note the altered bone marrow signal in the medial talar shoulder. There are no apparent morphological changes in the OLT cartilage. **c** The red overlay indicates the segmentation mask of the OLT cartilage, carefully avoiding the cortical bone and synovial fluid. Representative T1ρ maps (**d**) at rest, (**e**) with axial loading, and (**f**) traction. Note the decreased T1ρ values induced by 500 N axial load and increased T1ρ values induced by 120 N traction. The blue color indicates low T1ρ values and the red color indicates high T1ρ values. HC, Healthy controls; OLT, Osteochondral lesion of the talus

*In situ* axial loading led to a significant decrease in T1ρ values in visually intact cartilage regions of OLT patients (mean difference -1.1 ms; *p* = 0.030) and in matched cartilage areas of HC (mean difference -2.2 ms, *p* = 0.003), but not in the OLT cartilage (mean difference -1.3 ms, *p* = 0.150; Fig. 4). Further, *in situ* axial traction resulted in a significant T1ρ increase in visually intact cartilage regions of OLT patients (mean difference 1.4 ms, *p* = 0.030) and matched cartilage of HC (mean difference 2.3 ms, *p* = 0.030), but not in the OLT cartilage (mean difference 1.9 ms, *p* = 0.150; Figs. 4, 5). Axial loading did not induce significant changes in T1ρ values in the OLT cartilage in different OLT grades (grade 1, *p* = 0.750; grade 2, *p* = 0.918; grade 3, *p* = 0.160; and grade 4, *p* = 0.999). Similarly, there were no significant differences between OLT grades during axial traction (grade 1, *p* = 0.750; grade 2, *p* = 0.453; grade 3, *p* = 0.129; and grade 4, *p* = 0.999).

## Discussion

In this cross-sectional study, we investigated T1ρ relaxation times in the ankle joint at rest, with *in situ* axial loading and traction in patients with osteochondral lesions of the talus and healthy control subjects. At rest, we found significantly higher T1ρ relaxation times in the OLT cartilage compared to visually intact cartilage regions within the same patients and matched cartilage of HC. However, there was no significant difference in T1ρ values in the OLT cartilage between the different OLT grades. Following axial loading and traction, the OLT cartilage yielded consistently higher T1ρ relaxation times compared to the visually intact cartilage regions of OLT patients. Axial loading led to a significant decrease in T1ρ relaxation times in visually intact cartilage regions, but not in the OLT cartilage itself. Further, traction resulted in a significant increase in T1ρ values in visually intact cartilage regions, but not in the OLT cartilage itself.

In early cartilage degeneration, an inverse correlation between T1ρ relaxation times and relative proteoglycan concentration is well described in the current literature [11, 12, 30, 31]. In line with previous studies that assessed T1ρ relaxation times in degenerated cartilage of the knee, this study found increased T1ρ relaxation times in injured cartilage regions in patients with OLT [32–34]. However, given the previously shown distinct biochemical, cellular, and biomechanical properties in healthy and damaged cartilage of the knee and ankle, a direct comparison of these results may be limited [35, 36]. In this study, the T1ρ values of the OLT cartilage and matched HC cartilage showed significant overlap. Furthermore, no significant differences were observed in the OLT cartilage between different OLT grades. These findings indicate that T1ρ relaxation mapping may serve as a complementary tool rather than providing robust stratification of cartilage damage in the OLT alone.

T1ρ mapping may not only reflect cartilage composition but also its biomechanical properties. Changes in relaxation time in response to axial loading have been described as a surrogate for mechanical resilience with potential for *in vivo* monitoring of cartilage function [18–20, 37]. In this study, *in situ* axial loading induced a significant decrease in T1ρ relaxation times in visually intact cartilage regions, but not in the OLT cartilage itself. These findings might reflect the relative increase in the proteoglycan concentration occurring due to reductions in tissue thickness and water content during axial loading [38]. Furthermore, load-induced changes in T1ρ relaxation times might be accounted for by changes in collagen fiber orientation and hydration of hyaline cartilage [39]. In accordance, several studies reported decreased T1ρ relaxation times in cartilage samples during acute loading [37, 40]. In contrast to the present findings, others reported more pronounced changes in T1ρ relaxation times in early degenerative than in intact cartilage [37, 39]. However, the knee and ankle joints have different responses to load profiles, *e.g.*, due to differences in the articular surface configuration and cartilage stiffness, which limits the comparability of the results across the knee and ankle joints [35]. Our results indicate important differences between healthy and damaged cartilage in the OLT. However, the load-induced changes in healthy cartilage regions and the OLT showed significant overlap. Based on these findings, T1ρ relaxation mapping alone in response to axial loading may not provide a sufficient basis for differentiating early cartilage degeneration in the OLT cartilage.

In contrast to the widespread use of mechanical loading to quantitatively assess altered cartilage biomechanical properties, *in situ* axial traction in peripheral joint MR imaging has rather been used to improve diagnostic accuracy in morphological cartilage evaluation [41–44]. The present study investigated the impact of *in situ* axial traction on quantitative T1ρ relaxation times and found a traction-induced increase in T1ρ values in visually intact cartilage regions, but not in the cartilage covering the OLT. The underlying cartilage matrix changes remain unknown even though these findings may indicate that loading-unloading-induced nutrition of the cartilage is impaired. Moreover, axial traction may result in increased T1ρ relaxation times due to increased cartilage water content and relative reduction in proteoglycan content [11].

Quantitative MRI studies investigating whole ankle cartilage degeneration in patients with OLT are scarce. Thus far, only one study has evaluated quantitative cartilage compositional properties before therapeutic intervention using T2 mapping [45]. Marik et al reported elevated T2 values in cartilage areas of OLT compared to morphologically intact adjacent cartilage and cartilage of healthy controls [45]. The T1ρ relaxation mapping performed in this study, assessed compositional and biomechanical cartilage properties in OLT, as T1ρ mapping has been reported to be more reproducible under *in situ* axial loading compared to T2 mapping [25]. Furthermore, T1ρ mapping has been described as a more sensitive method than T2 mapping for detecting early changes in OA due to its high sensitivity to cartilage proteoglycan content [46, 47]. In agreement with Marik et al [44], the current study found increased T1ρ relaxation times in the OLT cartilage compared to visually intact cartilage regions of the affected ankle and matched cartilage of HC.

Chronic ankle instability is linked to OLT [21, 22], which in turn may alter ankle biomechanics, cause chronic inflammatory symptoms, and worsen its instability if OLT becomes unstable [48, 49]. Furthermore, the progression of cartilage injury in OLT is associated with abnormal biomechanical characteristics of the damaged cartilage. The interaction between damaged cartilage and the surrounding healthy cartilage areas in the progression of OLT is not yet understood. Biomechanical performance and biochemical homeostasis seem to interact. Ruan et al reported altered mechanical stress distribution in the surrounding cartilage area of the OLT defect region using finite element analysis [50]. However, their analysis only focused on a small area of adjacent cartilage. In contrast to these findings, significant differences in T1ρ relaxation times between visually intact cartilage regions of OLT patients and matched cartilage of HC were not observed in the present study. Moreover, this finding was independent of axial loading or traction.

This study has several limitations. First, these results are limited by the relatively small sample size of patients with OLT, which is, however, comparable to most previous studies of this kind. Larger cohorts in multi-center settings of this rare disease should be designed to increase the robustness of these findings. Secondly, histopathological analysis of the cartilage was not performed and, as surgery was not always indicated, correlation with arthroscopic diagnosis of cartilage pathology was not possible. Therefore, it is challenging to determine the exact cartilage composition covering the OLT. Due to the subchondral bone involvement in the OLT, a natural tissue injury response may occur, ultimately leading to fibrocartilage formation [51, 52]. There are notable differences in T1ρ values comparing hyaline and fibrocartilage, which may explain the different relaxation profiles in the OLT cartilage and intact cartilage regions of OLT patients and HC [53]. There are anatomic region-dependent differences in T1ρ relaxation times [28]. Due to the small sample size, pooled data from OLTs located both on the medial and lateral talar shoulder was included in the final analysis. This may have affected the overall error of the analysis. To achieve a more comprehensive assessment of the whole osteochondral unit, further studies should investigate the bone cartilage crosstalk. In addition, a separate analysis of deep and superficial cartilage layers was not performed. Several studies reported distinct response-to-loading patterns in the superficial and deep layers of the knee [20, 25, 54]. At the ankle joint, differentiation between cartilage layers is not feasible due to the small talar cartilage thickness and the limited spatial resolution of the MRI data. This study included relatively short rest periods before *in situ* axial loading. Different load-unload regimes are known to influence cartilage relaxation properties, which may account for the discrepancies when comparing our results to the current literature [55–57]. The repeatability of the T1ρ mapping sequence used in this study was evaluated in the knee cartilage of two healthy individuals. Compositional MRI of knee cartilage may not be directly comparable to that of the talus, particularly the OLT cartilage.

In conclusion, we showed that increased T1ρ relaxation times may serve as a sensitive imaging biomarker of early degenerative changes of hyaline cartilage in patients with OLT. Furthermore, the lack of load- and traction-induced T1ρ changes in OLT cartilage compared with visually intact cartilage regions may reflect altered biomechanical properties of damaged hyaline cartilage.

### Supplementary Information

ELECTRONIC SUPPLEMENTARY MATERIAL

## Data Availability

The experimental dataset of the current study is not publicly available to preserve individuals’ privacy under the European General Data Protection Regulation. A subset of the healthy controls (17/29) was previously reported in our study that assessed T1ρ relaxation mapping in individuals with chronic ankle instability. This dataset is available at the following URL: https://pubmed.ncbi.nlm.nih.gov/35611813/. This study focused on ankle instability and did not include any individuals with osteochondral lesions of the ankle joint and did not report on *in situ* axial traction.

## Abbreviations

ANOVA: Analysis of variance
BMI: Body mass index
HC: Healthy control
ICC: Intraclass correlation coefficient
MRI: Magnetic resonance imaging
OA: Osteoarthritis
OLT: Osteochondral lesion of the talus
