# Supplementary material for: T1ρ relaxation mapping in osteochondral lesions of the talus: a non-invasive biomarker for altered biomechanical properties of hyaline cartilage?
Source: Eur Radiol Exp. 2024 Jul 24;8:83. doi: 10.1186/s41747-024-00488-4 (PMC11269556; doi:10.1186/s41747-024-00488-4)
Supplement: Supplementary file 1 — ELECTRONIC SUPPLEMENTARY MATERIAL [file 41747_2024_488_MOESM1_ESM.pdf]

T1ρ relaxation mapping in osteochondral lesions of the talus: a non-invasive biomarker for altered biomechanical properties of hyaline cartilage?

ELECTRONIC SUPPLEMENTARY MATERIAL

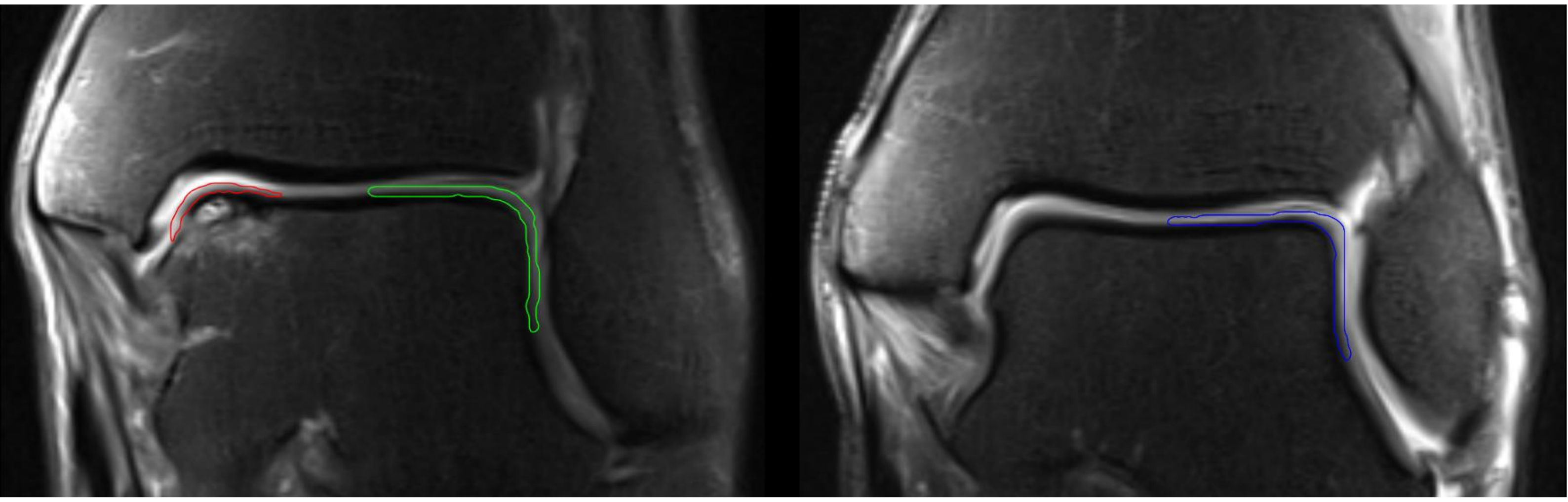

Supplemental Figure 1. Regional cartilage matching between OLT patients and healthy controls.

Representative regional cartilage matching between an OLT patient and a healthy control demonstrated on coronal PD-weighted fat-saturated images. Cartilage masks were drawn covering (a, red overlay) the OLT cartilage, (a, green overlay) the visually intact cartilage of the OLT patient, and (b, blue overlay) the cartilage of the healthy control. Note that visually intact cartilage in OLT patients, and healthy control cartilage, both contralateral to the OLT region were matched.
